# Endoscopic suture therapy for iatrogenic pharyngeal laceration: an extended application of endoluminal surgery

**DOI:** 10.1055/a-2719-3330

**Published:** 2025-11-06

**Authors:** Abdulrahman Qatomah, Harishankar Gopakumar, Daryl Ramai, Christopher C. Thompson

**Affiliations:** 1 1861 Gastroenterology, Hepatology, and Endoscopy, Brigham and Womenʼs Hospital, Boston, Massachusetts, United States; 2 37852 Division of Gastroenterology and Hepatology, King Faisal Specialist Hospital and Research Center, Jeddah, Saudi Arabia

Endoscopic suturing has emerged as a versatile therapeutic tool that extends the role of endoscopy beyond diagnostic and resectional interventions into advanced defect closure and tissue apposition. Using dedicated platforms such as the Overstitch (Boston Scientific, Marlborough, MA) system, full-thickness suturing can be performed through a flexible endoscope, enabling safe and durable closure of large mucosal defects, fistulas, anastomotic leaks, and perforations 1 2 . In addition, endoscopic suturing plays an expanding role in bariatric endoscopy, including revision of gastric bypass, primary endoscopic sleeve gastroplasty, and outlet reduction 3 4 . Compared to conventional closure techniques with clips or tissue sealants, suturing offers a stronger, more reliable, and often permanent closure, particularly in challenging anatomical locations 5 . With growing adoption worldwide, endoscopic suturing has become an essential skill in advanced endoscopy, bridging the gap between minimally invasive surgery and therapeutic endoscopy.

A 57-year-old female who suffered from weight regain following post-Roux-en-Y gastric bypass surgery with a body mass index of 32 kg/m ^2^ . The patient presented to outpatient endoscopy for a trans-oral reduction of the gastric outlet with endoscopic submucosal dissection (ESD-TORe). Following completion of ESD and argon plasma coagulation of the inner rim around the gastric outlet, the single-channel endoscope was withdrawn. We then advanced a double-channel scope mounted with an OverStitch device (Boston Scientific, Marlborough, MA) and advanced carefully to the oropharynx. However, a full-thickness laceration to the lateral portion of the soft palate was seen ( Fig. 1 **a, b** ). This was deemed related to a combination of pressure-induced trauma from the advancing gastroscope with a transparent cap and the Yankauer used to provide external suction. The decision was to proceed with endoscopic suturing. After applying the first suture, the visualization was suboptimal due to clotted blood, which was not removable despite water irrigation and scope suction. To avoid additional trauma from external suction, a through-the-scope suction was performed using a stent introduction catheter connected to wall suction ( Fig. 2 **a, b** ). This technique allowed for a precise, controlled intermittent suction and subsequent application of a second suture and cinching. Additional application of hemostatic agent to prevent bleeding and induce healing ( Video 1 ).

**Fig. 1:**
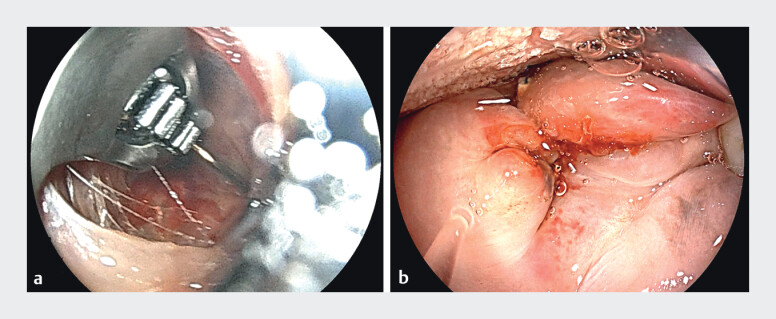
Endoscopic images of full-thickness laceration (left) and post-endoscopic closure (right).

**Fig. 2:**
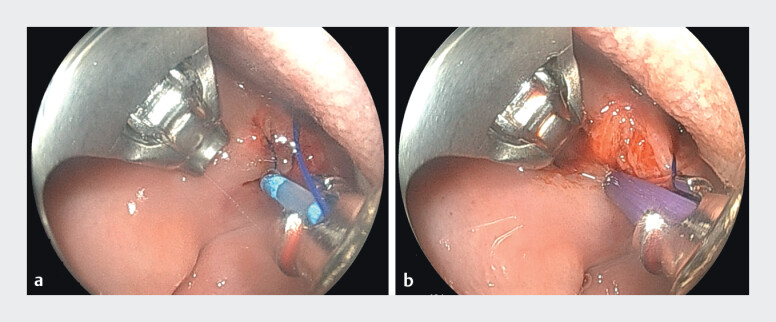
Endoscopic images of through-the-scope suction catheter (left) and hemostatic gel placement (right).

Endoscopic images of full-thickness laceration (left) and post-endoscopic closure (right).Video 1

The procedure was completed without further complications; the patient received antibiotic therapy, which was continued for 5 days, and was admitted for 24-hour observation. She developed oral pain post-procedure, which was managed with both intravenous pain medications and oral lidocaine. The pain subsided the following day, and the oropharyngeal exam revealed no remaining laceration. The patient was then discharged with a follow-up endoscopy in 2–4 weeks for suture removal.

Iatrogenic trauma to the oropharynx is common, and prompt recognition is a key to optimal management. Our case provides a novel application of endoscopic suture therapy in the oropharyngeal cavity; however, careful attention and precise suturing are crucial to avoid unnecessary injury to surrounding structures. To enhance visualization and avoid additional Yankauer-induced trauma, through the scope suction can be performed safely. Further studies are needed to confirm the efficacy of this novel application of endosuture therapy.

Endoscopy_UCTN_Code_TTT_1AO_2AO

## Notes

Endoscopy E-Videos https://eref.thieme.de/e-videos
*E-Videos* is an open access online section of the journal *Endoscopy* , reporting on interesting cases and new techniques in gastroenterological endoscopy. All papers include a high-quality video and are published with a Creative Commons CC-BY license. Endoscopy E-Videos qualify for HINARI discounts and waivers and eligibility is automatically checked during the submission process. We grant 100% waivers to articles whose corresponding authors are based in Group A countries and 50% waivers to those who are based in Group B countries as classified by Research4Life (see: https://www.research4life.org/access/eligibility/ ). This section has its own submission website at https://mc.manuscriptcentral.com/e-videos .

## References

[1] Kantsevoy SV Bitner M Mitrakov AA Endoscopic suturing closure of large mucosal defects after endoscopic submucosal dissection is technically feasible, fast, and eliminates the need for hospitalization Endosc Int Open 2014 2 E37 E40 10.1016/j.gie.2013.10.051 24332082

[2] Sharaiha RZ Kumta NA DeFilippis EM A large multicenter experience with endoscopic suturing for management of gastrointestinal defects and stent anchorage in 122 patients: a retrospective review J Clin Gastroenterol 2016 50 784 792 10.1097/MCG.0000000000000336 25984980

[3] Abu Dayyeh BK Rajan E Gostout CJ Endoscopic sleeve gastroplasty: a potential endoscopic alternative to surgical sleeve gastrectomy for treatment of obesity Gastrointest Endosc 2013 78 530 535 10.1016/j.gie.2013.04.197 23711556

[4] Jirapinyo P Thompson CC Endoscopic bariatric and metabolic therapies: surgical analogues and mechanisms of action Clin Gastroenterol Hepatol 2017 15 619 630 10.1016/j.cgh.2016.10.021 27989851 PMC5444453

[5] Stavropoulos SN Modayil R Friedel D Current applications of endoscopic suturing World J Gastrointest Endosc 2015 7 777 789 10.4253/wjge.v7.i8.777 26191342 PMC4501968

